# Oxabicyclic Guest Compounds as sII Promoters: Spectroscopic Investigation and Equilibrium Measurements

**DOI:** 10.3389/fchem.2020.00614

**Published:** 2020-07-17

**Authors:** Jiwoong Seol, Woongchul Shin, Juwoon Park

**Affiliations:** ^1^Faculty of Liberal Education, Seoul National University, Seoul, South Korea; ^2^Central Technology R&D Institute, Hyundai Oilbank Co., Ltd., Yongin-si, South Korea; ^3^Naval & Energy System R&D Institute, Daewoo Shipbuilding & Marine Engineering Co., Ltd., Siheung-si, South Korea

**Keywords:** sII promoter, oxabicyclic compounds, 1, 4-epoxycyclohexane, simple hydrate former, powder diffraction, NMR, Raman, gas storage

## Abstract

In this study, we investigate three oxabicyclic compounds, 3,6-dioxabicyclo[3. 1.0]hexane (C_4_H_6_O_2_, ETHF), 7-oxabicyclo[2.2.1]heptane (C_6_H_10_O, 14ECH), and 7-oxabicyclo[4.1.0]heptane (C_6_H_10_O, 12ECH) as novel promoters for gas hydrates. According to the outcomes of powder X-ray diffraction (PXRD) and synchrotron high-resolution powder diffraction (HRPD), all CH_4_ hydrates formed with ETHF, 14ECH, and 12ECH were identified to be sII (Fd-3m) hydrates with corresponding lattice parameters of 17.195, 17.330, and 17.382 Å, respectively. It was also clearly demonstrated that CH_4_ molecules are accommodated in the sII-S cages through solid-state ^13^C NMR and Raman spectra. Consequently, we clarified that the three compounds observed are large guest molecules (LGMs) that occupy the sII-L cages. Moreover, the thermodynamic stability of each LGM + CH_4_ (and N_2_) hydrate system was remarkably improved compared to that of the simple CH_4_ (and N_2_) hydrate. In particular, 14ECH manifested several unique features compared to the other two promoters. First, the 14ECH + CH_4_ hydrate did not dissociate up to room temperature (298 K), even at a moderate pressure of approximately 60 bar. At a given pressure, 14ECH increased the dissociation temperature of the CH_4_ hydrate by ~18 K, indicating that its promotion capability is as strong as that of tetrahydrofuran (THF), currently considered to be the most powerful promoter. Second, more interestingly, it was revealed by further PXRD, NMR, and Raman analyses that 14ECH forms a simple sII hydrate in the absence of help gases. According to differential scanning calorimetry (DSC) outcomes, we revealed that the simple 14ECH hydrate dissociates at 270~278 K under ambient pressure. In addition to the thermodynamic stability, we also note that the 14ECH + CH_4_ hydrate presented a sufficiently high temperature of formation, requiring little additional cooling. Given these promising features, we expect that the 14ECH hydrate system can be adopted to realize hydrate-based technologies. We also believe that the LGMs introduced here have considerable potential to serve as alternates to conventional promoters and that they can be widely utilized in both engineering and scientific research fields.

## Introduction

Clathrate hydrate, consisting of a hydrogen-bonded water framework and guest molecules, is now a well-known type of inclusion compound. Because general types of clathrate hydrates are mainly composed of water, various potential for sustainable hydrate-based technologies have long been suggested, including CH_4_/H_2_/natural gas storage (Florusse et al., 2004; Seo et al., 2009; Veluswamy et al., 2016), CO_2_ capture and storage (Park et al., 2006; Shin et al., 2008), gas separation (Seo et al., 2005; Babu et al., 2015), desalination (Cai et al., 2016), air-conditioning (Nakajima et al., 2008), and their use of functional materials (Yeon et al., 2008; Park et al., 2009; Shin et al., 2009; Seol et al., 2012). Meanwhile, versatile compounds have also been extensively investigated as co-guest additives. Such additional guest molecules are referred to as large guest molecules (LGMs) or large molecule guest substances (LMGSs) given their larger sizes compared to other gaseous guest compounds. An important purpose of employing LGMs, especially given the engineering and industrial applications of hydrates, is to promote the conditions for gas hydrate formation and preservation so as to minimize the total amount of energy consumed when utilizing hydrates. In most cases, though the thermodynamic stability levels are enhanced by incorporating promoters, the structure of the original CH_4_ hydrate (sI) is necessarily transformed to sII or sH, leading to reduced storage capacities by least 15~20%. However, many researchers have focused more on the thermodynamic stability, which is improved significantly, especially when adding sII promoters.

In terms of the guest distribution, most sII gas hydrates containing LGMs are classified as “double” hydrates, because the large (sII-L) and small (sII-S) cages are primarily occupied by LGMs and gaseous components, respectively. On the other hand, gas-free sII hydrates stabilized by a LGM component alone should be classified as a “simple” hydrate, and we simply refer to these LGMs as “simple sII formers” or “simple formers” in this paper. In general, gas hydrates bearing simple sII formers exhibit considerably higher thermodynamic stability levels compared to those formed with ordinary LGMs. For example, under pressure of approximately 40 bar, the dissociation temperature of furan + CH_4_ (~295 K, Pahlavanzadeh et al., 2016) or tetrahydrofuran (THF) + CH_4_ (~297 K, Lee et al., 2012) is considerably higher than that of the pyrrolidine + CH_4_ hydrate (~287 K, Shin et al., 2012). As an another example, at nearly 80 bar, the stable region of the 1,3-dioxane + CH_4_ hydrate (~297 K, Li et al., 2020) is much larger than that of the cyclohexane (CH) + CH4 (~290 K, Sun et al., 2002) hydrate. Moreover, simple hydrates of LGMs are expected to have a variety of applications, as they do not require pressurized gases and have good flexibility (i.e., tuning) of the guest compositions (Nakajima et al., 2008; Park et al., 2009; Seo et al., 2009; Shin et al., 2012). However, although numerous LGMs have been proposed over nearly a century, only a few of them have been found to be simple sII formers, apart from halogenated organics such as chlorofluorocarbons (CFCs). To the best of our knowledge, no new simple sII former has been discovered since Udachin et al. (2002) reported the structure of the simple hydrate of tetrahydropyran (THP) through single crystal diffraction.

It is very interesting that many the simple sII formers are empirically oxacyclic hydrocarbons such as propylene oxide, trimethylene oxide, 1,3-dioxolane, furan, THF, 1,3-dioxane, and tetrahydropyran (THP). In view of this, in this study, we investigate the three oxabicyclic compounds of 3,6-dioxabicyclo[3.1.0]hexane (C_4_H_6_O_2_), 7-oxabicyclo[2.2.1]heptane (C_6_H_10_O), and 7-oxabicyclo[4.1.0]heptane (C_6_H_10_O) as novel formers of sII gas hydrates. Instead of the IUPAC names, we will use corresponding synonyms of epoxytetrahydrofuran (ETHF), 1,4-epoxycyclohexane (14ECH) and 1,2-epoxycyclohexane (12ECH) for simplicity and unity in this paper. The rest of this paper describes the following step-by-step research. First, the crystal structure of each LGM + CH_4_ hydrate was determined by crystallographic analyses. Second, the distribution of the guest compounds was monitored through solid-state ^13^C NMR and Raman spectrometry. Third, the equilibrium P-T conditions of the LGM + CH_4_ (and N_2_) hydrate systems were measured to estimate the promotion performances. Fourth, further PXRD, NMR, Raman and DSC assessments were conducted and the results analyzed in detail to determine whether the promoters act as simple sII formers. Finally, the unique features of the 14ECH hydrate system focusing on the formation conditions were discussed.

## Experimental

H_2_O (LC-MS grade, Merck), ETHF (97%, Tokyo Chemical Industry), 14ECP (98%, Alfa Aesar), and 12ECH (98%, Tokyo Chemical Industry) and were used as received. High-purity CH_4_ (99.95%) and N_2_ (99.99%) were supplied by Daesung Industrial Gas Corp. of Korea. Compared to the stoichiometric composition (x_LGM_ = 0.0556) for the sII hydrate, approximately 5% excessive amount of each LGM was mixed with water and charged in a high-pressure resistance cell (V~100 ml). Then CH_4_ was supplied into the vessel until the initial pressure was reached 60~65 bar at ambient temperature (80~90 bar of N_2_ were supplied for N_2_ hydrate samples). The cell in each case was gradually cooled at the rate of −1 K∙h^−1^ with stirring maintained at 200 ± 10 RPM. The temperatures at which the pressure starts to drop varied significantly depending on the type of the LGMs used. However, we cooled the vessel to a sufficiently low temperature of approximately 260 K to obtain a sample in a fully solidified state. The vessel was then quenched by liquid nitrogen and the solid sample was quickly collected. Finally, for the subsequent spectroscopic analyses, it was ground into a fine powder (d <200 μm) inside liquid nitrogen.

To identify the crystal structures, we first utilized synchrotron high-resolution powder diffraction (HRPD) and powder X-ray diffraction (PXRD). For ETHF and 12ECH hydrates, the HRPD patterns were obtained with a single wavelength of 1.5216 Å, recording in a 2θ range of 5.0–126.0° (step width = 0.01° and scan time = 0.7 s/scan). For 14ECH and CH hydrates, the PXRD patterns were obtained with a Rigaku D/MAX-2500 equipment using a light source of Cu radiation with a wavelength of 1.54180 Å (Kα_1_ = 1.54056 Å and Kα_2_ = 1.54439 Å) and with a power of 8 kW (40 kV and 200 mA), recording in a 2θ range of 5.0–55.0° (step width = 0.02° and scan time = 1 s/scan). Every measurement temperature was kept at 150 K and no dissociation of the samples was detected. The crystal structures and corresponding lattice parameters were determined using FullProf and Checkcell programs.

Next, both solid-state NMR and Raman experiments were carried out to monitor the molecular behaviors of the guest components. Solid-state ^13^C NMR (HPDEC) experiments were conducted with the Bruker 400 MHz Avance II solid-state NMR at the Korea Basic Science Institute. We used a Larmor frequency of 100.4 MHz, a pulse length (p1) of 1.6 μs, and a repetition delay time (d1) of 3 s. The static signal of tetramethylsilane was referred to as 0 ppm at room temperature. All samples were measured at 210 K with a magic-angle spinning rate of 5 kHz. The vibration frequencies of the guest molecules were studied with high-resolution Raman equipment (Horiba Jobin Yvon LabRam HR Evolution). A 532-nm laser at 50 mW was used as an excitation source. All Raman spectra were obtained at 123 K with a low-temperature Linkam accessory.

To investigate the thermal properties of hydrates, temperature-dependent phase patterns were measured with differential scanning calorimeter (DSC) equipment (NETZSCH DSC 200 F3 Maia). A small piece of each sample (approximately 3~5 mg) was placed in aluminum pan, immersed in liquid nitrogen. The pan was then mounted on the sample stage that was precooled at 173 K. After the 10 min of isothermal time at 173 K and ambient pressure, the sample was slowly heated at a constant rate of 3 K/min to reach 298 K. During the overall measurement period, the sample stage was purged by nitrogen gas to prevent the humidity.

We also measured the equilibrium P-T conditions of the CH_4_ (or N_2_) hydrate systems containing the three LGMs. Each liquid mixture (total mass ~7 g) consists of a stoichiometric composition of LGM and balanced water was charged into a high-pressure resistance cell (V ~ 100 ml) and pressurized at various CH_4_ or N_2_ pressures at ambient temperature. The samples were continuously cooled to 260 K (at a rate of −1 K∙h^−1^) to form a solid hydrate phase. They were then more slowly heated to 310 K (at a rate of 0.3 K∙h^−1^). The stirring speed was kept at 200 ± 10 RPM during the overall hysteresis process. To verify the accuracy of our measurements, several equilibrium points of the simple CH_4_ and THF + CH_4_ hydrates were additionally measured. We note that our measurements demonstrated very good agreement with the reported values (Adisasmito et al., 1991; Lee et al., 2012).

## Results and Discussion

**Figure 1:**
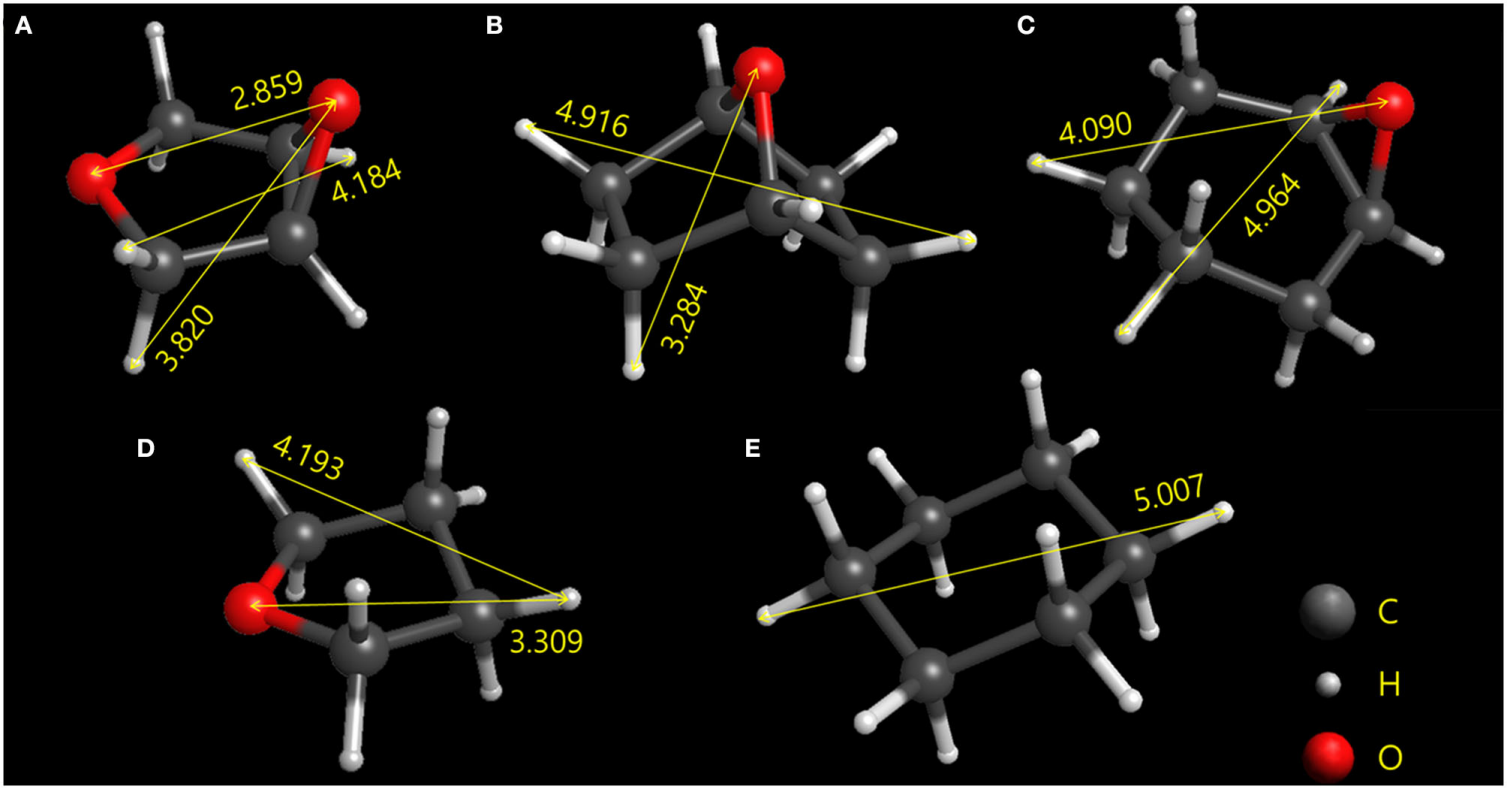
Optimized geometries of the **(A)** ETHF, **(B)** 14ECH, **(C)** 12ECH, **(D)** THF, and **(E)** CH. The longest center-to-center distances between each type of atom are given in angstroms.

The geometries and center-to-center distances of five molecules, ETHF, 14ECH, 12ECH, THF, and CH, are illustrated in Figure 1. The molecular geometries were optimized via B3LYP calculations with the basis set of 6-31G++ (d, p) using Gaussian 03 program (Frisch et al., 2004). The size of THF was in good agreement with the reported value (Takeya et al., 2018). There are two types of COC bonds: those in the epoxide rings and those in the five-membered rings. In the epoxide rings, both ETHF and 12ECH show similar CO bond lengths of 1.44 Å and COC bond angles of 61.5°. However, the five-membered rings of ETHF and 14ECH showed somewhat different geometries. The CO bond length and COC bond angle of ETHF were 1.43 Å and 110°, respectively, nearly identical to those of THF. However, the COC bond angle of 14ECH was found to be 96.6°, leading to a somewhat smaller CC distance of 2.16 Å compared to that of ETHF (2.35 Å). Consequently, the six-membered ring of 14ECH is more distorted and slightly smaller than that of 12ECH. Considering that the sizes of ETHF, 14ECH, and 12ECH are similar to those of THF and CH, one can expect that the three LGMs will form sII hydrates.

**Figure 2:**
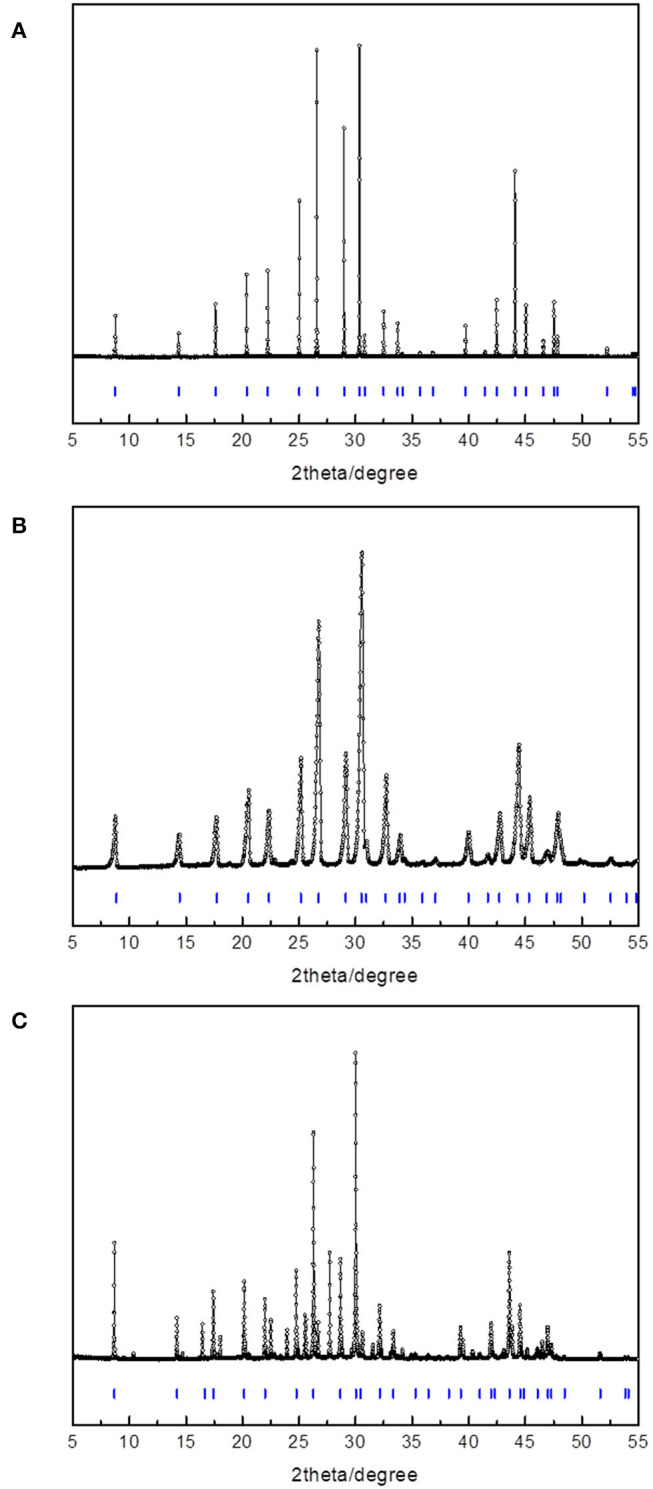
HRPD and PXRD patterns of the **(A)** ETHF, **(B)** 14ECH, and **(C)** 12ECH + CH_4_ hydrates.

Figure 2 and Table 1 shows the powder diffraction patterns and corresponding Miller indices of the CH_4_ hydrates formed with ETHF, 14ECH, and 12ECH. The structures of the ETHF, 14ECH, and 12ECH + CH_4_ hydrates were clearly identified to be the sII (Fd-3m) type with lattice parameters of 17.195, 17.330, and 17.382 Å, respectively. These lattice parameters correspond to unit-cell volumes of 5083.8, 5204.7, and 5251.89 Å^3^. The amount of hexagonal ice (Ih) or CH_4_ hydrate (sI) due to the exclusion of LGMs was minute or negligible. We also measured the powder diffraction pattern of the CH + CH_4_ hydrate and obtained a lattice parameter of a = 17.480 Å (Figure S1 and Table S1). The lattice parameter of the THF + CH_4_ hydrate was previously reported as a = 17.224 Å (Lee et al., 2012). Thus, the unit cell sizes are in the following order: ETHF < THF <14ECH <12ECH < CH + CH_4_ hydrates. Accordingly, we can conclude that the effective van der Waals volume of the >CH–O–CH < group is slightly smaller than that of the -CH_2_-CH_2_- group.

**Table 1:** Miller indices of the three hydrates shown in Figure 2.

**ETHF** **+** **CH**_****4****_			**14ECH** **+** **CH**_****4****_			**12ECH** **+** **CH**_****4****_		
**Peaks**	***hkl***	***d_***hkl***_***	**Peaks**	***hkl***	***d_***hkl***_***	**Peaks**	***hkl***	***d_***hkl***_***
8.79	111	9.928	8.84	111	10.006	8.696	111	10.0355
14.38	202	6.079	14.46	202	6.127	14.223	202	6.1455
17.63	222	4.964	16.97	311	5.225	16.694	113	5.2409
20.39	400	4.299	17.73	222	5.003	17.442	222	5.0177
22.24	133	3.945	20.50	400	4.333	20.166	400	4.3455
25.04	422	3.510	22.36	313	3.976	21.997	133	3.9877
26.58	333	3.309	25.17	422	3.538	24.764	422	3.5481
28.99	404	3.040	26.73	333	3.335	26.292	333	3.3452
30.35	513	2.907	29.15	404	3.064	28.671	404	3.0727
30.79	424	2.866	30.52	513	2.929	30.015	513	2.9381
32.50	602	2.719	30.96	424	2.888	30.451	424	2.897
33.73	335	2.622	32.68	602	2.740	32.141	206	2.7483
34.13	226	2.592	33.92	335	2.643	33.359	533	2.6507
35.70	444	2.482	34.32	622	2.613	35.305	444	2.5089
36.84	515	2.408	35.90	444	2.501	36.429	515	2.434
39.74	355	2.239	37.05	515	2.427	38.239	426	2.3228
41.46	800	2.149	38.89	426	2.316	39.291	355	2.2629
42.47	337	2.101	39.96	355	2.256	40.994	800	2.1727
44.10	822	2.026	41.69	800	2.166	41.988	733	2.1235
45.06	555	1.986	42.71	337	2.117	42.315	446	2.1079
46.63	408	1.923	43.04	644	2.102	43.603	822	2.0485
47.54	537	1.887	44.35	822	2.042	44.55	555	2.0071
47.85	248	1.876	45.32	555	2.001	44.862	266	1.9938
52.24	755	1.728	45.64	626	1.988	46.094	408	1.9434
54.48	737	1.662	46.89	408	1.938	47.001	537	1.9079
54.75	666	1.655	47.82	537	1.902	47.301	248	1.8965
			48.12	824	1.891	48.484	646	1.8529
			49.33	646	1.847	51.634	755	1.747
			50.22	913	1.817	53.841	737	1.6804
			51.68	844	1.769	54.112	666	1.6726
			52.54	755	1.742			
			53.96	268	1.699			
			54.79	737	1.675			

**Figure 3:**
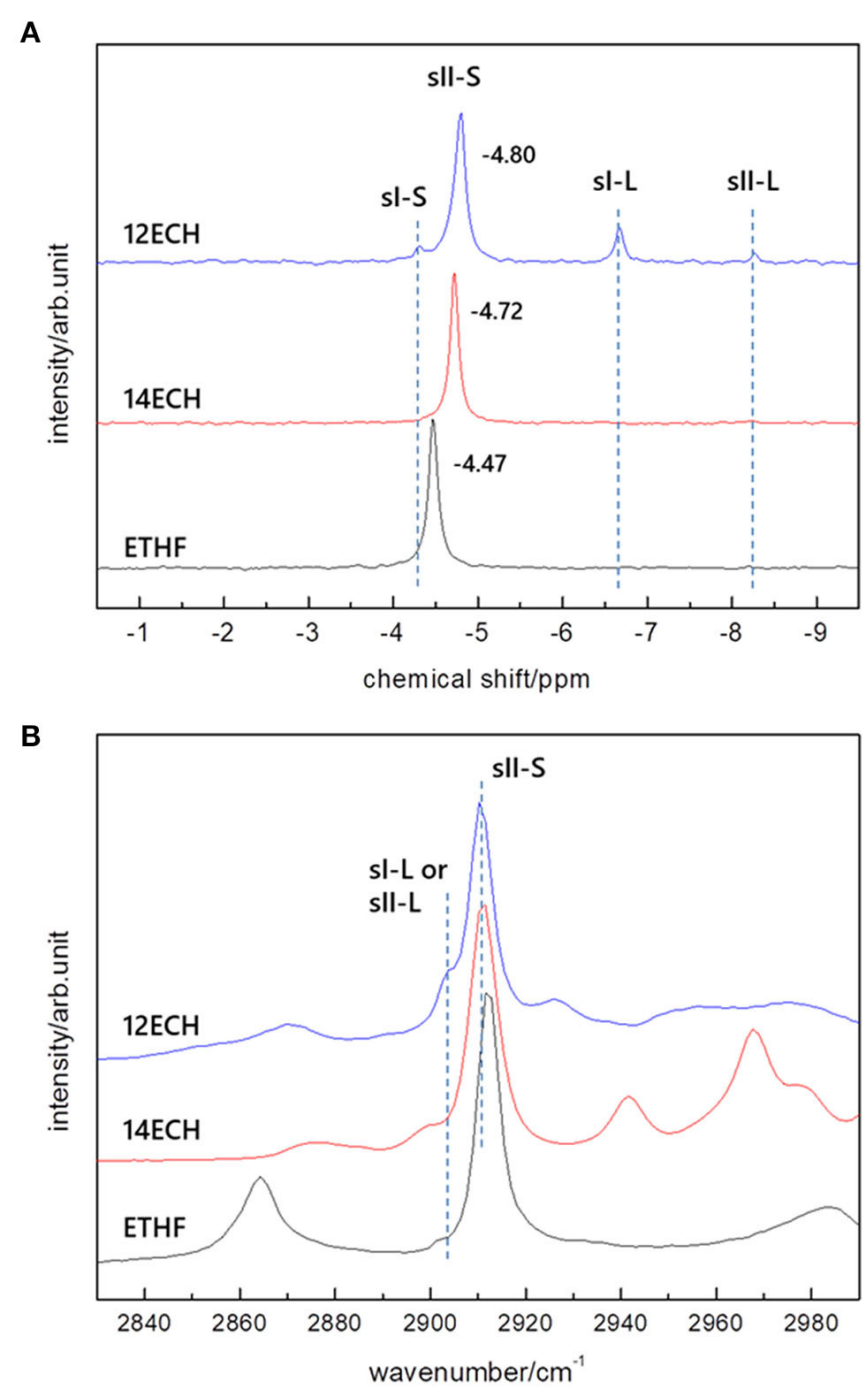
**(A)** solid-state ^13^C NMR and **(B)** Raman spectra of the ETHF, 14ECH, and 12 ECH + CH_4_ hydrates.

Solid-state ^13^C NMR and Raman spectroscopy were utilized in order to cross-check the structures and monitor the CH_4_ guest molecules. As shown in Figure 3A, a single clear peak at δ = −4.5 ~ −4.8 ppm was detected for every CH_4_ hydrate, corresponding to the CH_4_ molecule entrapped in the small (5^12^) cage. No other peaks were detected for the ETHF and 14ECH + CH_4_ hydrates in the upfield region. Although additional signals at −4.3, −6.7, and −8.3 ppm, typical of CH_4_ molecules entrapped in sI-S, sI-L, and sII-L cages (Yeon et al., 2006), respectively, were observed for the 12ECH + CH_4_ hydrate, these peaks are quite small compared with the main peak near −4.8 ppm. The Raman spectra in Figure 3B allow the same interpretation; C-H stretching vibration bands near 2,911 cm^−1^ were clearly observed for all samples, indicative of a CH_4_ molecule in a small cage, whereas only a weak signal near 2,902 cm^−1^ (sI-L or sII-L) was additionally detected for the ECH + CH_4_ hydrate. To summarize the outcomes of the HRPD, ^13^C NMR, and Raman investigations, all LGMs can readily form “double” sII hydrates with CH_4_, of which the large and small cages are mainly occupied by LGM and CH_4_ molecules, respectively. Moreover, the ^13^C NMR peaks found at −4.47 (ETHF), −4.72 (ECH), and −4.80 ppm (ECH) clearly indicated that even when the type of the enclosing cage is identical (sII-S), the CH_4_ molecule in the larger cage exhibits a more shielded chemical shift. It is not a great difference; however, the propensity is clear enough such that one can roughly estimate which hydrate has a larger lattice parameter by comparing the NMR peaks without precise crystallographic analyses.

**Figure 4:**
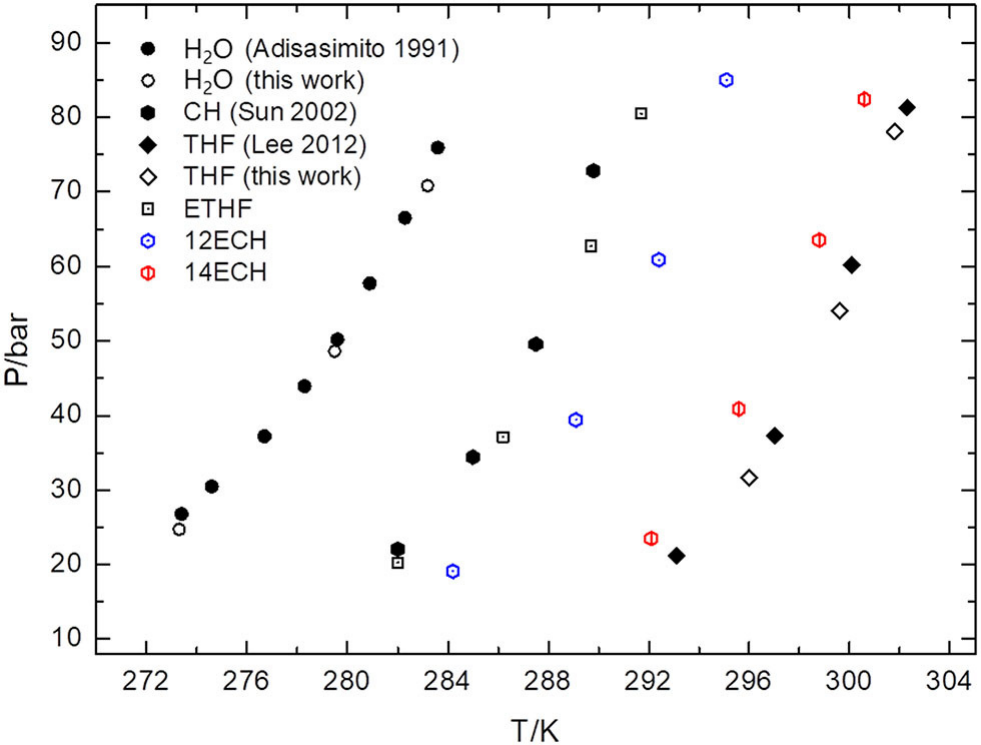
Equilibrium P-T conditions of CH_4_ hydrates containing various promoters (x_LGM_ = 0.0556).

Next, we measured the equilibrium P-T conditions of the three CH_4_ hydrate systems at a constant mole fraction of the liquid phase (x_LGM_ = 0.0556) and listed the results in Table 2. By incorporating each LGM, the thermodynamic region of the CH_4_ hydrate was significantly expanded to higher temperature and lower pressure level. At a given pressure, the dissociation temperatures of the simple CH_4_ hydrate (Adisasmito et al., 1991 and this work) were increased by nearly 9, 12, and 18 K due to the addition of ETHF, 12ECH, and 14ECH, respectively. In particular, the 14ECH + CH_4_ hydrate did not dissociate up to room temperature (298 K), even under a moderate pressure of ~60 bar. To compare the degree of the promotion clearly, we illustrate the equilibrium conditions of the CH_4_ hydrates containing CH (as a moderately strong promoter) and THF (as a particularly strong promoter) as well in Figure 4. Comparing the promoters in similar molecular structures, the promotion effect of ETHF (black empty square) is much weaker than that of THF (Lee et al., 2012 and this work). On the other hand, compared to the CH + CH_4_ hydrate (Sun et al., 2002), the 12ECH and 14ECH + CH_4_ hydrates (blue and red hexagons, respectively) showed higher dissociation temperatures. Moreover, the thermodynamic stability of the 14ECH + CH_4_ hydrate was found to be comparable to that of the THF + CH_4_ hydrate. It is notable that the promotion performance of 14ECH is as strong as that of THF, to the best of our knowledge, because THF is known to be one of the most powerful promoters.

**Table 2:** Equilibrium P-T conditions of CH_4_ hydrates containing three promoters.

**ETHF**		**14ECH**		**12ECH**	
**T (K)**	**P (bar)**	**T (K)**	**P (bar)**	**T (K)**	**P (bar)**
282.0	20.3	292.1	23.5	284.2	19.1
286.2	37.1	295.6	40.9	289.1	39.4
289.7	62.7	298.8	63.5	292.4	60.9
291.7	80.5	300.6	82.4	295.1	85.0

**Figure 5:**
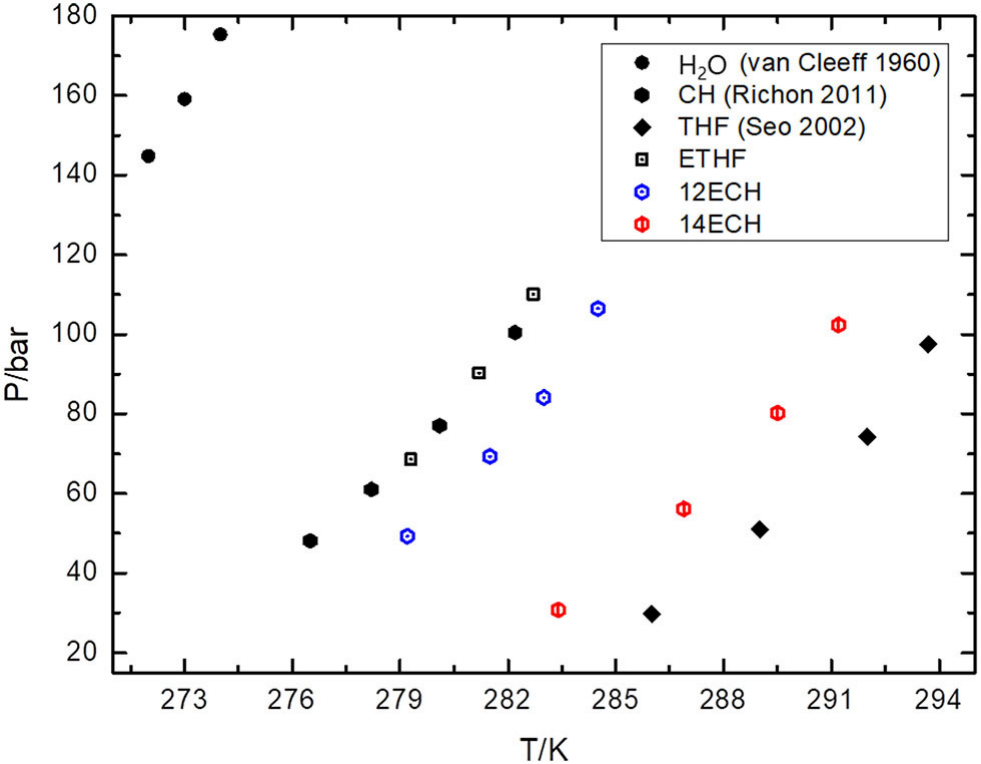
Equilibrium P-T conditions of N_2_ hydrates containing various promoters (x_LGM_ = 0.0556).

**Figure 6:**
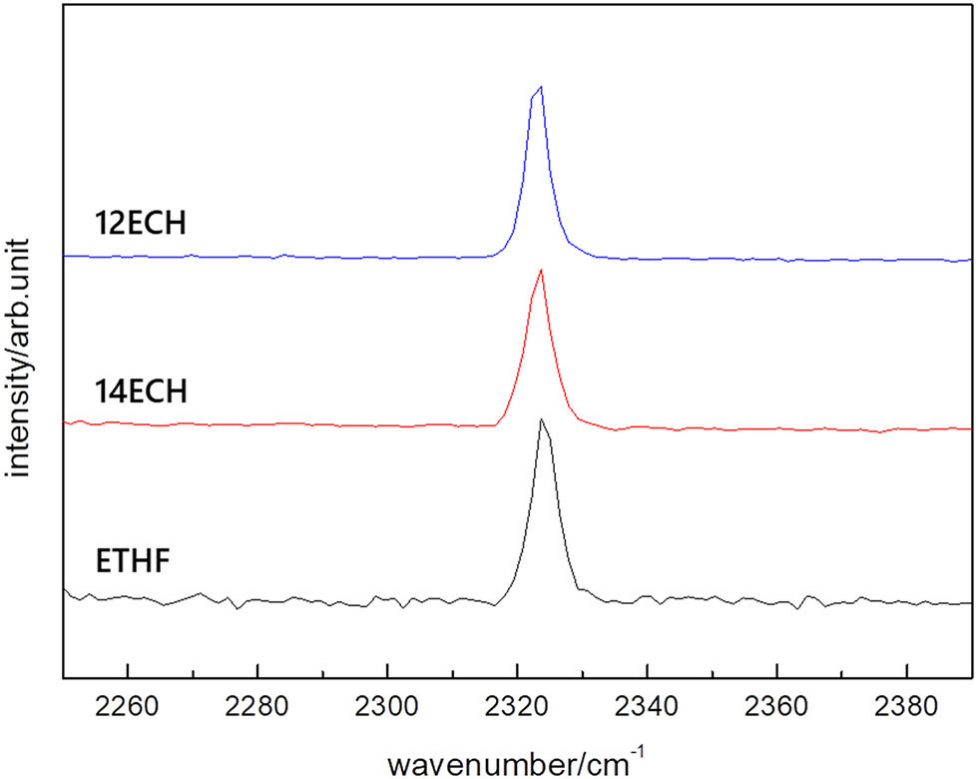
Raman spectra of the LGM + N_2_ hydrates measured at 123 K.

We also examined the equilibrium conditions of the three LGM + N_2_ hydrates and listed the results in Table 3. The addition of the LGMs dramatically improved the dissociation conditions of the simple N_2_ hydrate (van Cleeff and Diepen, 1960) to higher temperature and lower pressure levels. All promoters tended to be identical as the CH_4_ hydrate systems in terms of the degree of the promotion (Figure 5). For the CH + N_2_ and THF + N_2_ hydrates, we correspondingly referred to Richon and Mohammadi (2011) and Seo et al. (2001). To verify the inclusion of N_2_ in the solid hydrate phase, we utilized Raman spectroscopy. In Figure 6, the peaks near 2,324 cm^−1^ indicate that all LGM + N_2_ hydrates were clearly formed. Accordingly, we can conclude that the three novel LGMs serve as promoters for gas hydrates of both spherical and cylindrical types of gaseous molecules.

**Table 3:** Equilibrium P-T conditions of N_2_ hydrates containing three promoters.

**ETHF**		**14ECH**		**12ECH**	
**T (K)**	**P (bar)**	**T (K)**	**P (bar)**	**T (K)**	**P (bar)**
279.3	68.6	278.4	30.8	279.2	49.3
281.2	90.2	286.9	56.2	281.5	69.3
282.7	110.1	289.5	80.2	283.0	84.1
		291.2	102.4	284.5	106.5

**Figure 7:**
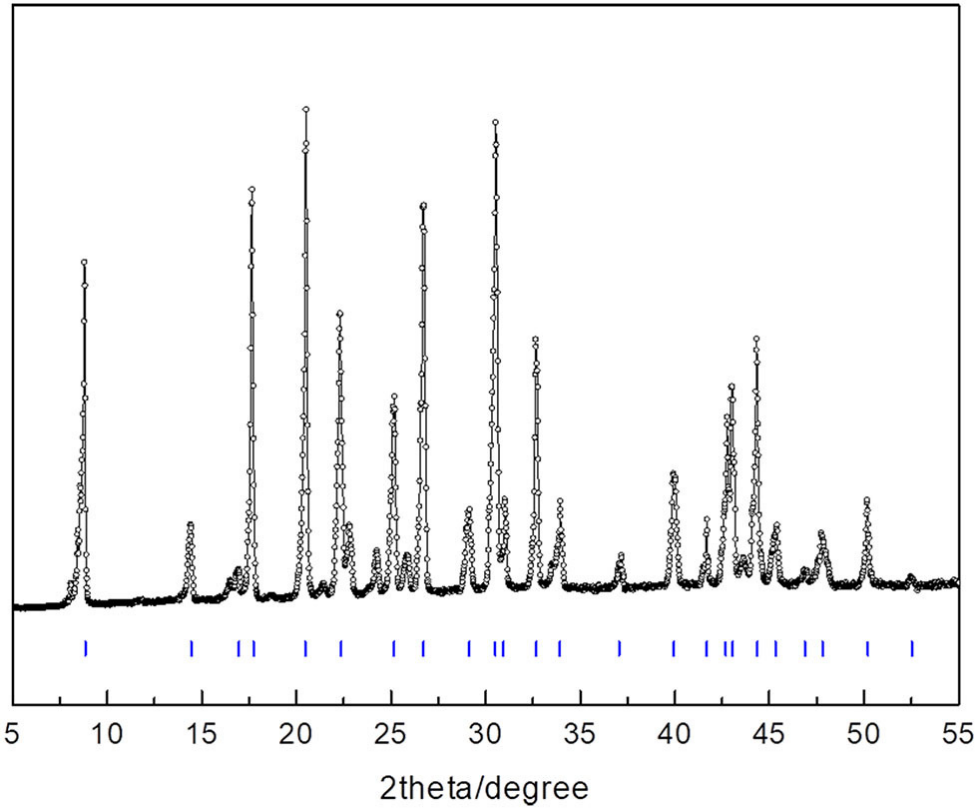
PXRD pattern of the 14ECH + H_2_O hydrate (gas-free) at 150 K.

To find clues by which to understand the powerful promotion of 14ECH, we examined samples that consist only of H_2_O and LGMs. Each liquid mixture of LGM + H_2_O was slowly cooled from the ambient temperature under continuous stirring to prepare a sufficiently homogeneous solid sample. According to crystallographic analyses, no hydrate phases were observed in the solid samples of ETHF + H_2_O and 12ECH + H_2_O. On the other hand, the PXRD pattern (Figure 7 and Table 4) clearly demonstrated that 14ECH + H_2_O forms a simple sII hydrate even in the absence of additional gaseous components. The lattice parameter was determined to be 17.333 Å at 150 K, practically identical to that of the 14ECH + CH_4_ hydrate. The inclusion of CH_4_ molecules in the sII-S cages appears to have little effect on the lattice size.

**Table 4:** Miller indices of the simple 14ECH hydrate shown in Figure 7.

**Peaks**	***hkl***	***d_***hkl***_***
8.84	111	10.007
14.45	202	6.128
16.97	311	5.226
17.73	222	5.004
20.50	004	4.333
22.36	133	3.977
25.17	422	3.538
26.72	333	3.336
29.14	404	3.064
30.51	315	2.930
30.95	424	2.889
32.67	602	2.741
33.91	335	2.643
35.89	444	2.502
37.04	515	2.427
38.88	426	2.316
39.95	355	2.257
41.69	008	2.167
42.70	337	2.118
43.03	644	2.102
44.34	822	2.043
45.31	555	2.001
46.88	048	1.938
47.81	357	1.903
49.32	466	1.848
50.21	913	1.817
51.67	844	1.769
52.53	557	1.742

**Figure 8:**
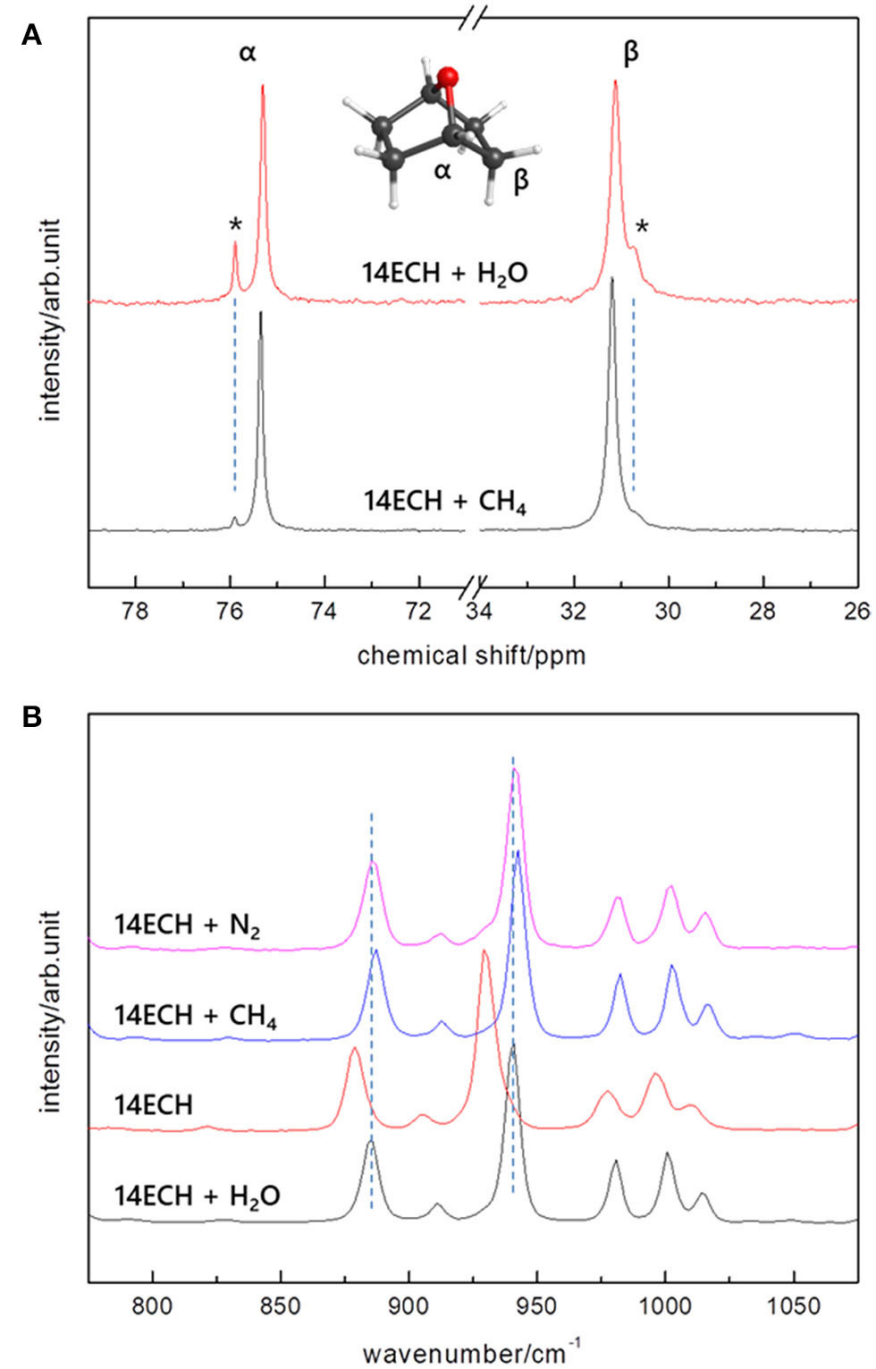
**(A)** ^13^C NMR peaks and **(B)** COC vibration bands of the 14ECH molecules.

To monitor the 14ECH molecules involved in the sII phase clearly, we analyzed the ^13^C NMR and Raman spectra in detail. The ^13^C NMR peaks of 14ECH molecules are shown in Figure 8A. For the 14ECH + CH_4_ hydrate (blue), it was clearly found that the alpha carbons of 14ECH exhibited two separate peaks at 75.3 and 75.9 ppm. The minute peak at 75.9 ppm should be attributed to 14ECH excluded from the hydrate phase, as we introduced a slightly excessive amount of 14ECH compared to the stoichiometric composition (by ~5%). Consequently, it can be seen that the main peak at 75.3 ppm, being also observed in the simple hydrate of 14ECH, originates from the 14ECH molecules entrapped in the sII-L cages. We also investigated the Raman spectra in detail, focusing on the vibration bands of the 14ECH molecules. In Figure 8B, several peaks near 850~1,000 cm^−1^ are due to symmetric stretching vibrations of the COC group (Socrates, 2001). However, for a comparison with the bands from pure 14ECH (red), those from the 14ECH accommodated in the sII phase clearly appeared at higher wavenumbers. As a free 14ECH molecule becomes confined in the sII-L cage, the COC bonds would contract slightly to increase the force constant of the vibration. In addition, the shortened bond length would increase the electron density around the alpha carbon to provide a more shielded condition. Spectroscopic measurements have thus far been exclusively utilized to monitor the gaseous guest component. However, our outcomes demonstrate convincingly that NMR and Raman spectra are also informative when seeking to determine the behaviors of LGMs.

**Figure 9:**
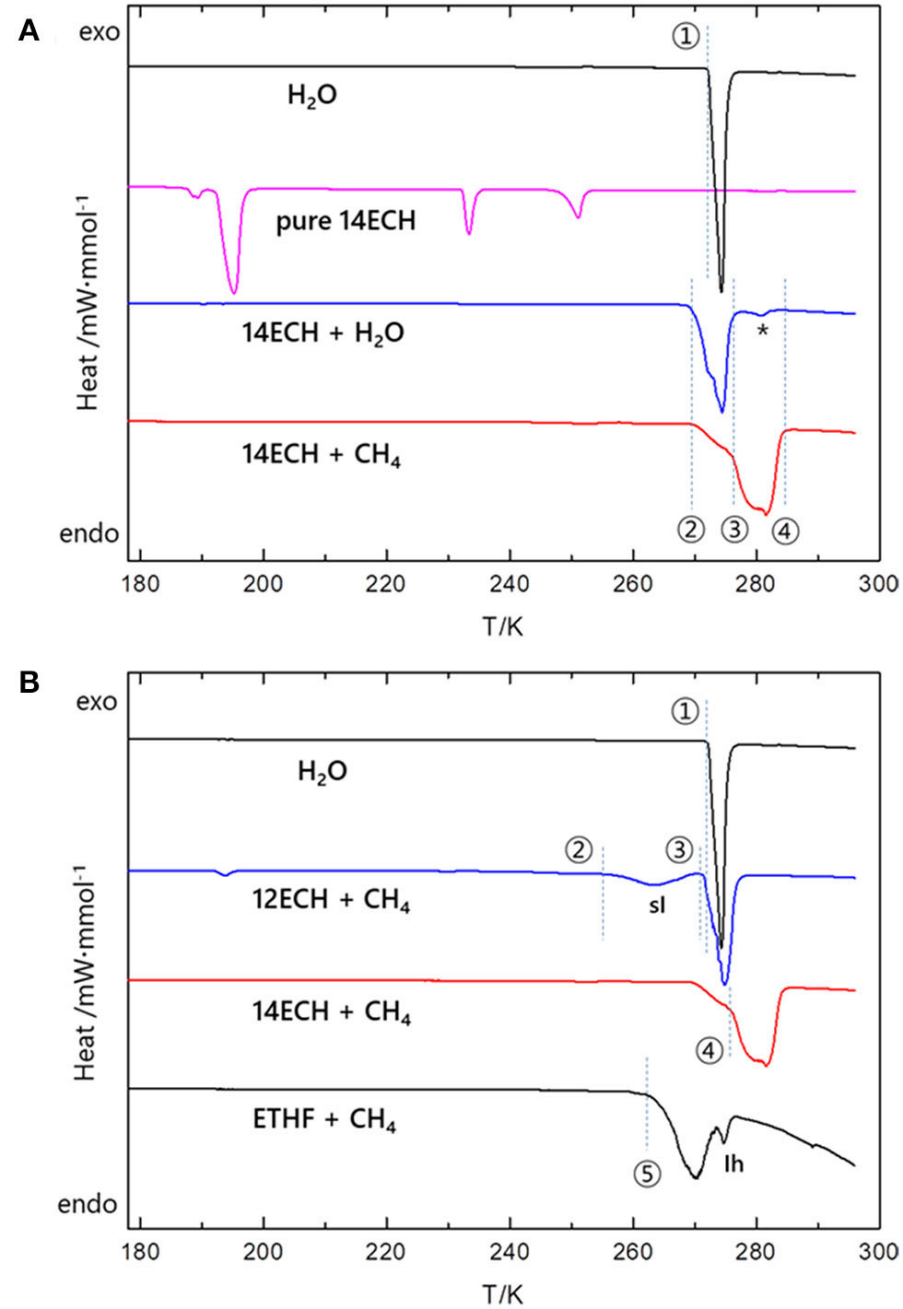
Several principal phase transitions induced by temperature at ambient pressure. **(A)** Four points ①~④ are correspondingly 273, 270, 277, and 285 K. A small peak that may be attributed to the 14ECP + air hydrate (impurity) is denoted by asterisk (*). For clarity, y axis is shown in the same scale. The original heat flows measured in the unit of mW·mg^−1^ were normalized by the mole of H_2_O (black, blue, and red). For pure 14ECH, the heat flow was normalized by the mole of 14ECH (magenta). **(B)** The five points ①~⑤ at which the phase transitions start to occur are correspondingly 273, 255, 271, 277, and 263 K. The heat flows were normalized by the mole of H_2_O.

In addition, the temperatures for the 14ECH hydrate systems at which the principal phase transitions occur were identified by differential scanning calorimetry (DSC) measurements. As seen in Figure 9A, the simple 14ECH hydrate starts to dissociate at 270 K and fully dissociates at 278 K. For the 14ECH + CH_4_ hydrate sample, two phase transitions were mainly observed: (1) the dissociation of a small amount of the 14ECH simple hydrate that are unreacted with gaseous CH_4_ (point ②); and (2) the dissociation of the 14ECH + CH_4_ hydrate induced by gas release (point ③). Eventually, the 14ECH + CH_4_ hydrate fully collapses at approximately 285 K (point ④). Meanwhile, the temperature-induced pattern of pure 14ECH is quite complex that we cannot determine its corresponding phase transitions at the present stage. However, the phase transitions induced by pure 14ECH were observed neither in the 14ECH + CH_4_ hydrate nor in the 14ECH simple hydrate. It provides another clear evidence of complete enclathrations of 14ECH molecules in both simple and CH_4_ hydrates. In addition, we can conclude that the simple 14ECH hydrate dissociates in the range of 270~278 K, whereas the 14ECH + CH_4_ hydrate dissociates in the range of 277~285 K at ambient pressure. In Figure 9B, both the onset and end temperatures of gas hydrate dissociations are in the following order: ETHF + CH_4_ <12ECH + CH_4_ <14ECH + CH_4_. This is fully consistent with the result of the phase equilibria shown in Figure 4.

Thus far, we have demonstrated that 14ECH acts as a simple sII former and that it appears to play a key role in the outstanding promotion effect. Out of the numerous LGMs proposed over the decades, simple hydrate formers are quite rare, except for CFCs. Furthermore, to the best of our knowledge, tetrahydropyran (THP) with a van der Waals diameter of 6.95 Å (Udachin et al., 2002) has been recorded as the largest simple sII former. However, on the basis of our calculation, 14ECH with the longest end-to-end distance of 7.10 Å is larger than THP. [Because the van der Waals radii are not included in Figure 1, to obtain the end-to-end distances we should add 1.09 and 1.56 Å to the H and O atoms, respectively (Rowland and Taylor, 1996)]. This is also supported by the fact that the lattice parameter of the 14ECH + H_2_O hydrate is somewhat larger than that of the THP + H_2_O hydrate (a = 17.289 Å at 153 K) (Takeya et al., 2018). Thus, we believe that this is the first finding of a simple sII former larger than THP.

**Figure 10:**
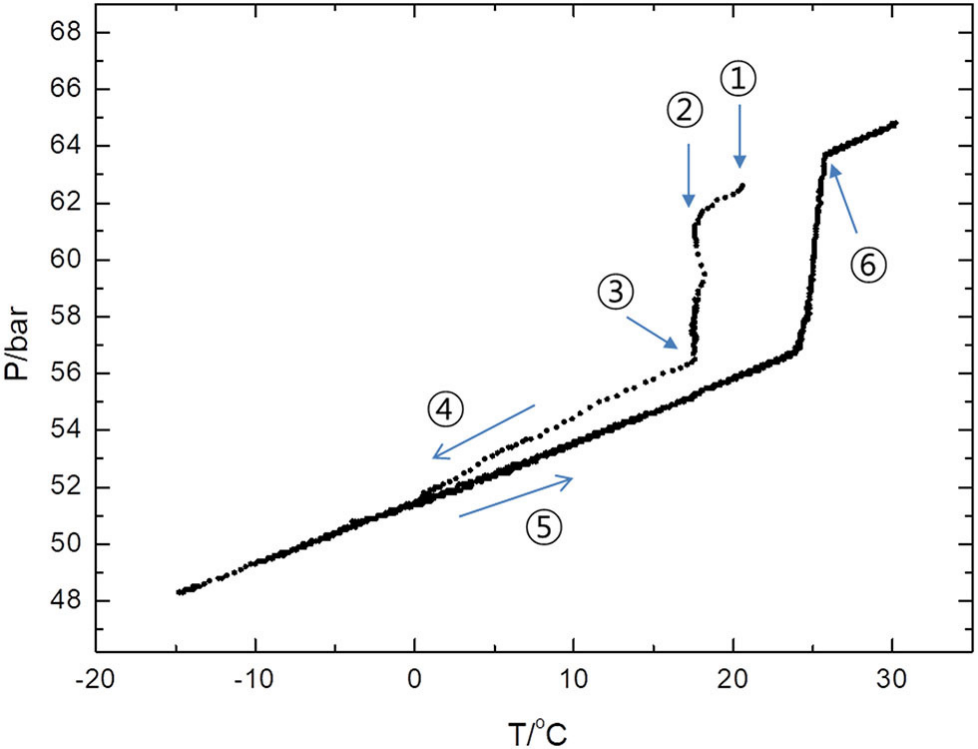
P-T trajectory created by the formation and dissociation processes of the 14ECH + CH_4_ hydrate. ① Upon the completion of the gas injection, the temperature inside the vessel was increased to 20°C by adiabatic compression. ② Within 10 min, the vessel was recovered to the ambient temperature (17.5°C) and CH_4_ uptake was initiated at the same time. ③ After 40 min, the vessel was immersed in an ethanol bath. ④ The vessel was cooled with a rate of −20 K/h. ⑤ The vessel was heated at a rate of +0.3 K/h. ⑥ The hydrate was fully dissociated at 25.7°C and 63.5 bar.

Finally, we noted that CH_4_ uptake takes place rapidly at a significantly high temperature by adding 14ECH. Figure 10 shows a P-T trajectory created by the formation and dissociation processes of the 14ECH + CH_4_ hydrate. It was clearly observed that the pressure began to drop sharply at the ambient temperature to form the hydrate phase. The high temperature of gas uptake may also be attributed to the capability of 14ECH to stabilize the simple hydrate. When the mixture of LGM + H_2_O is pressurized with the desired gas, theoretically the hydrate must form just under the equilibrium temperature. Owing to kinetic limitations in most cases, however, one has to cool the systems far below the equilibrium temperature to initiate gas uptake. Accordingly, a sufficiently high temperature of formation is necessary for industrial applications, because the additional time, energy, and cost requirements for further cooling and mixing processes would become considerable. Because the kinetics outcomes strongly depend on the highly various conditions under which the hydrate is formed, including the cell dimensions, initial amounts of the liquid and gas, contact area, stirring rate, and cooling rate, among other factors, diverse studies must of course be further carried out to investigate the detailed kinetic behaviors. At the present stage, however, it is still noteworthy that one can form the 14ECH + CH_4_ hydrate at ambient temperatures under a moderate pressure level.

## Conclusions

In this study, CH_4_ hydrates containing the three novel LGMs of ETHF, 14ECH, and 12ECH, were synthesized and characterized. According to the crystallographic and spectroscopic outcomes, all hydrates were identified to be sII (Fd-3m) double hydrates, of which the large and small cages were predominantly occupied by LGMs and gaseous molecules, respectively. The thermodynamic stability of each LGM + CH_4_ (and N_2_) hydrate was remarkably enhanced compared to that of the simple CH_4_ (and N_2_) hydrate. Particularly, 14ECH manifested several unique features compared to the other two promoters. First, on the basis of clear evidence from PXRD, NMR, Raman and DSC analyses, 14ECH was revealed to be a simple sII former with the largest size ever found. Second, 14ECH exhibited an excellent promotion effect, comparable to that of THF that is currently considered to be the most powerful promoter. In addition to the thermodynamic stability, the 14ECH + CH_4_ hydrate presented a sufficiently high temperature of formation, requiring little additional cooling. Given these promising features, we believe that the 14ECH hydrate system can be employed to facilitate hydrate-based technologies. However, because the present study focused mainly on fundamental characterizations of the structure and stability of the materials, for industrial applications, a variety of key engineering points should be studied in future works, including the capacities of gas storage, the kinetics of the gas uptake/release process, the stability of the promoters themselves, optimization of the conditions, and related areas. Finally, we would like to emphasize that the LGMs introduced here have considerable potential to serve as alternates to conventional promoters and that they can be widely utilized in both engineering and scientific research fields. We also believe that the findings and outcomes reported here have extended the fundamentals of clathrate hydrate, especially in relation to functional ice materials.

## Data Availability Statement

All datasets presented in this study are included in the article/Supplementary Material.

## Author Contributions

JS planned and managed the entire experiment, prepared and measured samples, analyzed data, produced the figures, and wrote up the draft. JS and WS measured and discussed HRPD data together. JS and JP discussed the outcomes of the equilibrium measurements together. All authors contributed to the article and approved the submitted version.

## Conflict of Interest

WS and JP were employed by the company Hyundai Oilbank Co., Ltd. and Daewoo Shipbuilding and Marine Engineering Co., Ltd., respectively. The remaining author declares that the research was conducted in the absence of any commercial or financial relationships that could be construed as a potential conflict of interest.
