# Supplementary figures and images for: Oxabicyclic Guest Compounds as sII Promoters: Spectroscopic Investigation and Equilibrium Measurements
Source: Front Chem. 2020 Jul 17;8:614. doi: 10.3389/fchem.2020.00614 (PMC7396542; doi:10.3389/fchem.2020.00614)

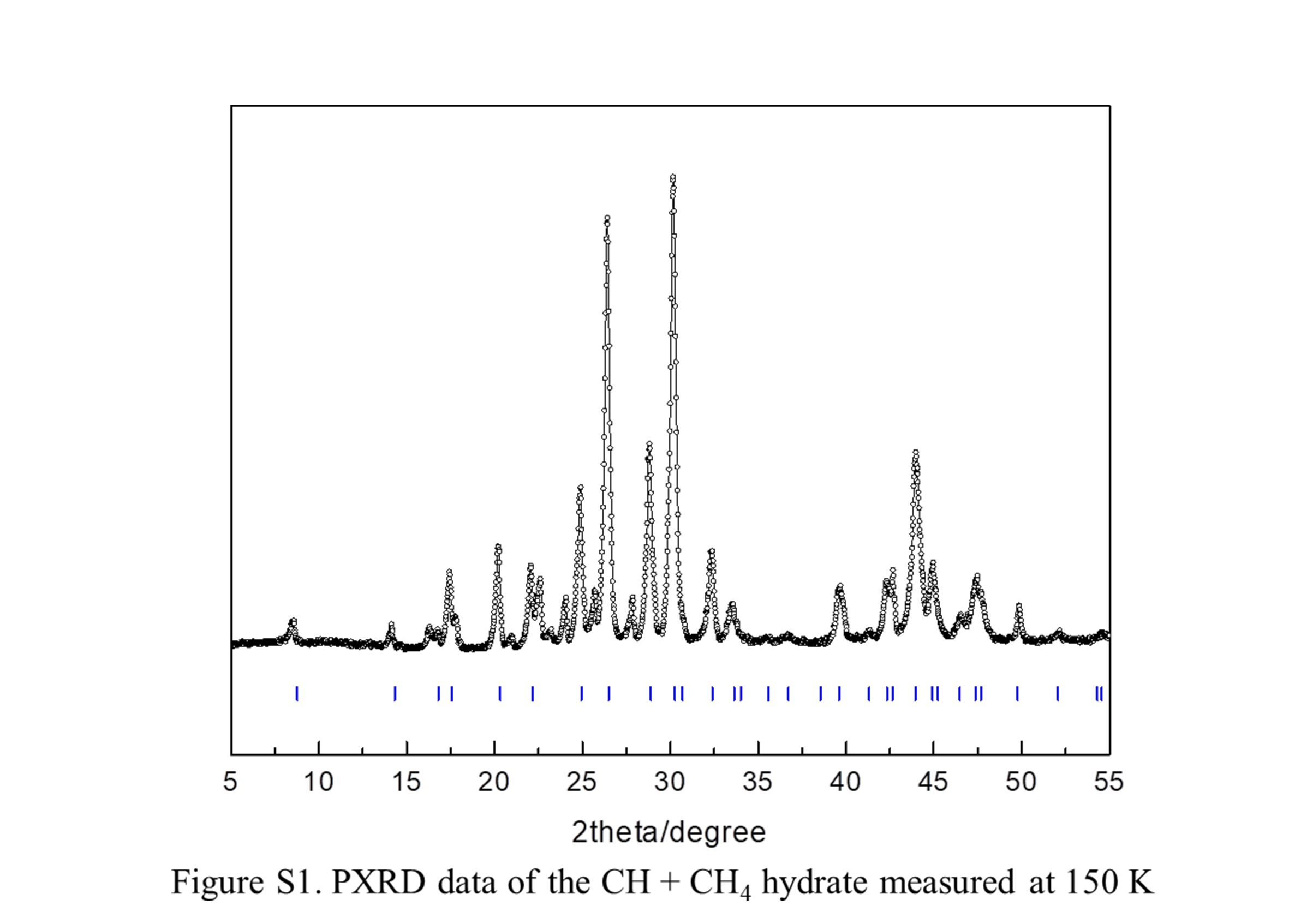

Supplement: Supplementary file 1 [file Image_1.TIF]
