# Supplementary material for: Oxabicyclic Guest Compounds as sII Promoters: Spectroscopic Investigation and Equilibrium Measurements
Source: Front Chem. 2020 Jul 17;8:614. doi: 10.3389/fchem.2020.00614 (PMC7396542; doi:10.3389/fchem.2020.00614)
Supplement: Supplementary file 2 [file Table_1.pdf]

Table S1. Miller Indices of the CH + CH_4 hydrate shown in Figure S1.

peaks	*h k l*	d_{hkl}
8.76	111	10.092
14.33	202	6.180
16.82	311	5.270
17.58	222	5.046
20.32	004	4.370
22.17	313	4.010
24.96	224	3.568
26.50	333	3.364
28.89	404	3.090
30.25	513	2.955
30.69	424	2.913
32.39	602	2.764
33.62	335	2.666
34.02	622	2.635
35.58	444	2.523
36.72	515	2.448
38.54	426	2.336
39.60	355	2.276
41.32	008	2.185
42.32	337	2.136
42.65	644	2.120
43.95	228	2.060
44.91	555	2.018
45.22	626	2.005
46.46	048	1.954
47.38	357	1.919
47.68	248	1.907
49.76	913	1.832
52.06	755	1.757
54.28	737	1.690
54.56	666	1.682
